# Supplementary figures and images for: Rosiglitazone alleviates LPS-induced endometritis via suppression of TLR4-mediated NF-κB activation
Source: PLoS One. 2024 Mar 28;19(3):e0280372. doi: 10.1371/journal.pone.0280372 (PMC10977739; doi:10.1371/journal.pone.0280372)

GAPDH 37KD

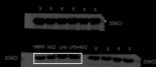

GAPDH 37KD

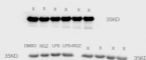

IkBa 39KD

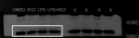

IkBa 39KD

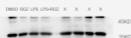

p65 65KD

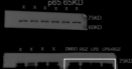

p65 65KD

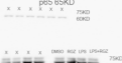

p-IkBa 40KD

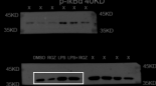

p-IkBa 40KD

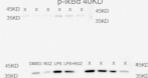

p-p65 65KD

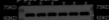

p-p65 65KD

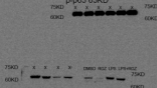

TLR4 96KD

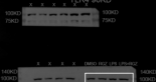

TLR4 96KD

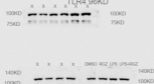

Supplement: S1 Raw images — (PDF) [file pone.0280372.s001.pdf]
