# Supplementary material for: Rosiglitazone alleviates LPS-induced endometritis via suppression of TLR4-mediated NF-κB activation
Source: PLoS One. 2024 Mar 28;19(3):e0280372. doi: 10.1371/journal.pone.0280372 (PMC10977739; doi:10.1371/journal.pone.0280372)
Supplement: Attachment — Submitted filename: recomendation.pdf [file pone.0280372.s003.pdf]

Recommendations:

1. In order to further confirm the effect of rosiglitazone on the Nf-kb pathway, it is recommended to add immunofluorescence staining to observe the nuclear translocation of Nf-kb.

2. It is recommended to add TLR4 inhibitors to observe whether they have the same protective effect.

3. It is recommended to detect the level of inflammatory factors in animal models, rather than judging the success of modeling by morphological changes of tissues.
