# Supplementary material for: Rosiglitazone alleviates LPS-induced endometritis via suppression of TLR4-mediated NF-κB activation
Source: PLoS One. 2024 Mar 28;19(3):e0280372. doi: 10.1371/journal.pone.0280372 (PMC10977739; doi:10.1371/journal.pone.0280372)
Supplement: Attachment — Submitted filename: Response to Reviews.doc [file pone.0280372.s002.doc]

**Response to reviews**

Dear Editors and Reviewers:

We greatly appreciate your and other reviewers' criticism and thoughtful suggestions. These valuable opinions will help improve the quality of the manuscript. We have carefully considered these opinions and made corresponding modifications, hoping for approval. The revised manuscript has been adjusted to the requirements of the journal. All changes made to the manuscript are highlighted in red for easy recognition. The following are the main corrections and responses to the reviewer's comments:

**Response to comments of Editor：**

1. The written should be improved and the grammar errors needed to be corrected.

**Response:** Thank you very much for your sincere suggestions. According to your suggestions, we have carefully revised the language format of the article and invited relevant professionals to review and correct it.

2. In Table 1, the primer is in human or in mouse, it needed to be clearly indicated in Table 1.

**Response:** Thank you very much for your careful review of this manuscript. In order for other readers to have a clearer understanding of the procedures of this study, we have clearly stated the source of primers in the manuscript according to your suggestion.

3. How do you design the dose of RGZ in in vivo studies?

**Response:** Thank you very much for your comments. As mentioned in the fifth line of “Animals and treatment” in the manuscript, we set the experimental dosage of this project with reference to previous relevant research reports.

4. TLR4 affected by RGZ demonstrated in this study. Whether RGZ affected other TLR family numbers, such TLR1, TLR2, etc？

**Response:** Thank you again for your careful reading and professional questions. Many studies have shown that Toll-like receptors (TLRs) are part of the innate immune system and participate in the response to microbial pathogens. It has been reported that normal skin epidermal keratinocytes compositively express TLR1, TLR2 recognizes a variety of ligands expressed by Gram-positive bacteria, and TLR3, TLR4 and TLR5 specifically recognize double-stranded RNA, Gram-negative lipopolaccharide and bacterial flagellin, respectively. In this study, endometritis was mainly caused by Gram-negative bacteria, so the TLR4 receptor was mainly studied. As for the other receptors you mentioned, this gives us an idea for other things we can do with RGZ in the future.

**Response to comments of Reviewer 1：**

The study is well designed and organized. The reviewer suggests that the manuscript be checked for the grammatical and typographical errors.

**Response:** Thank you very much for your valuable recommendations. We apologize for our grammatical errors and typographical errors. In accordance with the requirements of the journal, we carefully checked the grammar in the revised manuscript and asked professionals to make corrections.

**Response to comments of Reviewer 2：**

1. In the histopathologic results, the authors mentioned the histological scores, perhaps they should explain how they were calculated.

**Response:** Thank you very much for your suggestion. We are sorry for the trouble caused by our simple expression. Based on your professional advice, we have provided a detailed description of the grading criteria for histopathology score in the revised manuscript.

2. In WB results, the authors mentioned that “overexpression of TLR4 weakened the effect of RGZ on LPS-induced p65 phosphorylation and IκB expression in HESCs” which is not very understandable here.

**Response:** Thank you very much for your suggestion. We apologize for the confusion caused by our improper expression. We have rephrased the WB results in the revised manuscript to facilitate readers' clearer understanding of this mechanism.

3. The results for cell viability and cytokines production should be divided into two sections.

**Response:** Thank you very much for your valuable recommendations. We accepted your suggestion and divided cell viability and cytokines production into two parts in the revised manuscript.

4. In cytokines production results, the authors mention the protein expression of IL-1β and IL-6, where it is better to write the relative expression of genes. In addition, the authors said that consistent with the in vivo results, there is no in vivo results were seen.

**Response:** Thank you very much for your professional comments.

We accept your professional suggestions and have made modifications in the revised manuscript according to your suggestions. At the same time, we are sorry for your misunderstanding caused by our inappropriate description. In the revised manuscript, we restated this result to prevent confusion among other readers. We collected serum from mice and detected the expression of IL-1β and IL-6 using ELISA method β, but there was no statistically significant difference between the groups, possibly due to the lack of systemic response caused by local inflammation of the endometrium. This is consistent with the conclusion reported in the literature that acute phase protein assay has not proved useful in the diagnosis of endometritis[1]. Therefore, the results were not included in the manuscript.

[1] Sikora M , Król, Jaros aw, Nowak M ,et al.The usefulness of uterine lavage and acute phase protein levels as a diagnostic tool for subclinical endometritis in Icelandic mares.Acta Veterinaria Scandinavica, 2015, 58(1):50. DOI:10.1186/s13028-016-0233-4.

5. Fig.5 legend, mRNA should not be capitalized.

**Response:** Thank you very much for your advice. We have corrected this error in the revised manuscript.
