# Supplementary material for: Rosiglitazone alleviates LPS-induced endometritis via suppression of TLR4-mediated NF-κB activation
Source: PLoS One. 2024 Mar 28;19(3):e0280372. doi: 10.1371/journal.pone.0280372 (PMC10977739; doi:10.1371/journal.pone.0280372)
Supplement: Attachment — Submitted filename: Response to Reviewers.docx [file pone.0280372.s004.docx]

**Response to reviews**

Dear Editors and Reviewers:

We are delighted to receive your comments and suggestions from you and the reviewers. We have carefully considered these comments and made corrections accordingly, which we hope will be approved for publication. All changes made to the text are highlighted in green for easy identification. The main revisions and replies to the reviewer's comments are as follows:

**Response to comments of Reviewer 3：**

1. In order to further confirm the effect of rosiglitazone on the Nf-kb pathway, it is recommended to add immunofluorescence staining to observe the nuclear translocation of NF-κB.

**Response:** Thank you very much for your valuable suggestion. Based on your feedback, we have supplemented the results of immunofluorescence staining to observe the nuclear translocation of NF-κB and described them in the manuscript. The results are shown in Figure 7.

2. It is recommended to add TLR4 inhibitors to observe whether they have the same protective effect.

**Response:** Thank you for your valuable advice. We accept your proposal. We added this part of the experiment in the revised manuscript to ensure the reliability of the experimental results.

3. It is recommended to detect the level of inflammatory factors in animal models, rather than judging the success of modeling by morphological changes of tissues.

**Response:** Thank you very much for your valuable advice. We have supplemented this experiment in the experimental section of the manuscript to further ensure experimental rigor.
